# Efficacy of Unsupervised YouTube Dance Exercise for Patients With Hypertension: Randomized Controlled Trial

**DOI:** 10.2196/65981

**Published:** 2025-01-09

**Authors:** Mizuki Sakairi, Taiju Miyagami, Hiroki Tabata, Naotake Yanagisawa, Mizue Saita, Mai Suzuki, Kazutoshi Fujibayashi, Hiroshi Fukuda, Toshio Naito

**Affiliations:** 1 Department of General Medicine Faculty of Medicine Juntendo University Tokyo Japan; 2 Juntendo Advanced Research Institute for Health Science Tokyo Japan; 3 Medical Technology Innovation Center Juntendo University Tokyo Japan

**Keywords:** dance, video, exercise therapy, hypertension, blood pressure therapy, YouTube, mHealth

## Abstract

**Background:**

High blood pressure (BP) is linked to unhealthy lifestyles, and its treatment includes medications and exercise therapy. Many previous studies have evaluated the effects of exercise on BP improvement; however, exercise requires securing a location, time, and staff, which can be challenging in clinical settings. The antihypertensive effects of dance exercise for patients with hypertension have already been verified, and it has been found that adherence and dropout rates are better compared to other forms of exercise. If the burden of providing dance instruction is reduced, dance exercise will become a highly useful intervention for hypertension treatment.

**Objective:**

This study aims to investigate the effects of regular exercise therapy using dance videos on the BP of patients with hypertension, with the goal of providing a reference for prescribing exercise therapy that is highly feasible in clinical settings.

**Methods:**

This nonblind, double-arm, randomized controlled trial was conducted at Juntendo University, Tokyo, from April to December 2023. A total of 40 patients with hypertension were randomly assigned to either an intervention group (dance) or a control group (self-selected exercise), with each group comprising 20 participants. The intervention group performed daily dance exercises using street dance videos (10 min per video) uploaded to YouTube. The control group was instructed to choose any exercise other than dance and perform it for 10 minutes each day. The activity levels of the participants were monitored using a triaxial accelerometer. BP and body composition were measured on the day of participation and after 2 months. During the intervention period, we did not provide exercise instruction or supervise participants’ activities.

**Results:**

A total of 34 patients were included in the study (16 in the intervention group and 18 in the control group). The exclusion criteria were the absence of BP data, medication changes, or withdrawal from the study. The mean age was 56 (SD 9.8) years, and 18 (53%) of the patients were female. The mean BMI was 28.0 (SD 6.3) m/kg^2^, and systolic blood pressure (SBP) and diastolic blood pressure (DBP) were 139.5 (SD 17.1) mm Hg and 85.8 (SD 9.1) mm Hg, respectively. The basic characteristics did not differ between the two groups. In the multivariate analysis, SBP and DBP improved significantly in the intervention group compared to the control group (mean SBP –12.8, SD 6.1 mm Hg; *P*=.047; mean DBP –9.7, SD 3.3 mm Hg; *P*=.006).

**Conclusions:**

This study evaluated the effects of dance exercise on patients with hypertension, as previously verified, under the additional condition of using dance videos without direct staff instruction or supervision. The results showed that dance videos were more effective in lowering BP than conventional exercise prescriptions.

**Trial Registration:**

University Hospital Medical Information Network UMIN 000051251; https://center6.umin.ac.jp/cgi-open-bin/ctr_e/ctr_view.cgi?recptno=R000058446

## Introduction

High blood pressure (BP) is a major chronic disease that threatens people’s health and is an important risk factor for many types of heart, brain, and kidney vascular diseases. A total of 590,000 Japanese individuals with high BP continuously receive medical care, the highest number among lifestyle-related diseases [1]. The prevalence of high BP among adults in the United States was 29% from 2011 to 2014, and the prevalence rates increased with age: 18-39 years, 7.3%; 40-59 years, 32.2%; and 60 years and older, 64.9% [2]. The global population aged older than 65 years is expected to double between 2019 and 2050 [3]. Japan has the oldest population worldwide; in 2013, those aged older than 65 years exceeded 25% of the population and are expected to exceed 40% by 2060 [4]. Therefore, high BP is a global public health problem, and the number of patients with the condition is expected to increase with the growth of the aging population.

High BP is associated with an unhealthy lifestyle. The clinical treatment of high BP involves antihypertensive medications and lifestyle interventions, such as reducing salt intake, eating a diet rich in fruits and vegetables, exercising, and maintaining a healthy body weight [5]. Although antihypertensive medications are the main treatment, exercise is also an important recommendation for patients with high BP [6-8]. It is known that regular moderate exercises, such as water walking, brisk walking, running, small-sided soccer, and swimming, have beneficial effects on BP in patients with hypertension [9-13]. The World Health Organization recommends at least 150 minutes of moderate to vigorous physical activity (MVPA) per week [14]. However, in Japan, only about half of the population (59.6% of men and 46.9% of women) meets these physical activity standards [15]. Furthermore, during the COVID-19 pandemic, restrictions on outdoor activities led to decreased physical activity levels [16]. It has also been suggested that safety concerns, especially for women when exercising alone outdoors or after sunset, as well as fear of criticism, are barriers to engaging in physical activity [17]. Challenges in securing time and space for exercise due to caregiving, childcare, employment, and pandemics hinder physical activity. Furthermore, although physical activity interventions delivered or prompted by health professionals in primary care appear effective in increasing participation in MVPA, exercise prescription training for health care professionals is inadequate [18].

Dance, a fun form of exercise that uses music and can be performed in confined spaces, remains feasible, even in situations such as the COVID-19 pandemic. Dance was part of Japan’s educational curriculum in 2012 and was added as an Olympic sport starting in 2024 [19]. A survey conducted in Japan indicated that the proportion of teenagers participating in hip-hop dance at least once a week rose from 2.1% in 2015 to 3.5% in 2023 [20]. Therefore, dance has become an accessible sport, and compared to other activities such as marathon running or swimming, is easier for patients to perform in terms of space and time. A meta-analysis comparing dance to other exercises found that adherence and dropout rates for dance were better than those for other forms of exercise [21]. Previous studies have shown that regular dance therapy can benefit hypertension management in patients [22-30]. However, to the best of our knowledge, no studies in Japan have examined the effects of dance on BP. Additionally, previous studies involved direct patient monitoring during exercise or used internet-based methods for monitoring. In clinical settings, it is challenging to gather participants for regular prescribed group dance sessions or to monitor them using video chat. We, therefore, aimed to investigate the effect of regular dance therapy interventions on BP in patients with hypertension to provide a reference for prescription studies on dance exercise therapy in these patients. We hypothesized that performing the same movements without monitoring using self-made dance videos could lower BP and be useful as a nonpharmacological treatment for high BP.

## Methods

### Ethical Considerations

This study was approved by the Ethics Committee of Juntendo University (approval: E22-0387). The participants received written information about the trial, including its aim, expected advantages, and role, and were asked to provide written informed consent. This study was retrospectively registered with the University Hospital Medical Information Network (UMIN) under ID UMIN 000051251 and with the International Standard Randomized Controlled Trial Number registry (under ID ISRCTN46013). The UMIN is a network member of the Japan Primary Registries Network, as described in the World Health Organization registry network. All procedures were performed in accordance with relevant guidelines and regulations.

### Setting and Design

This study was conducted at the Juntendo University Department of General Medicine, Tokyo, Japan, a regional core hospital that treats many patients with lifestyle-related diseases. Outpatients generally visit the hospital every 2 months.

This was a nonblind, double-arm randomized controlled trial conducted from April 1, 2023, to December 27, 2023. Based on a previous study [31], we set the intergroup difference (difference from baseline) to –9 and the SD at 9. The results of previous studies are as follows: mean difference (MD) –8.75 mm Hg; 95% CI –6.51 to –10.39 for systolic BP, and MD –8.35 mm Hg; 95% CI –6.25 to –10.45 for diastolic BP. This study anticipated a similar decrease in BP, as reported previously. With a desired power of 80%, a sample size of 34 individuals was calculated. Considering a dropout rate of 15%, we selected a sample size of 40 participants, allocated in a 1:1 ratio into two groups using a random number table: the intervention (dance) group (n=20) and the control group (n=20). TM created the randomization table, staff members (MSakairi) conducted the recruitment, and the admin assistant conducted the group allocation.

We included outpatients with high BP from the Juntendo University Department of General Medicine. These patients with hypertension had been diagnosed with hypertension and were receiving regular oral medication. The patient was invited to participate in this study by their primary physician, whom they regularly visited for hypertension management, and consent was obtained. Participants were informed that their participation in this study was voluntary and that they could withdraw if they chose to discontinue after joining. Additionally, if their primary physician determined that withdrawal was necessary due to changes in their medical condition, the study could be terminated. We excluded patients with complications rendering them unsuitable for exercise, such as cardiovascular disease, cerebral vascular disease, those unable to balance on one leg, and patients who were newly prescribed antihypertensive drugs or who were administered antihypertensives later.

### Interventions

#### Development of Dance Videos

**Figure 1:**
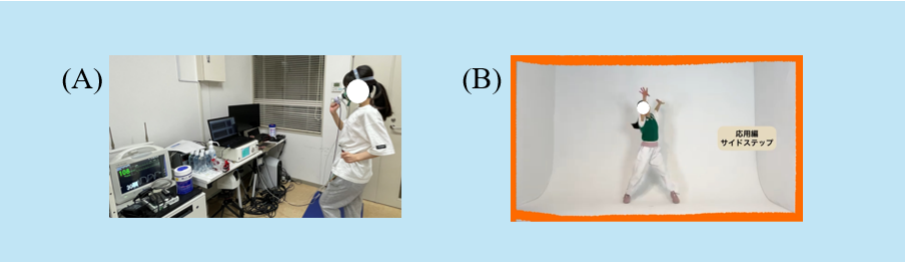
Details about dance. (A) The process of creating the dance. We have used exhaled breath analysis to measure the activity level of dance and created five videos ranging from 4.5 to 7 METs. (B) A part of the distributed dance video. We distributed the video of the dance we created to participants using YouTube. MET: metabolic equivalent of task.

The intervention group watched an approximately 10-minute-long dance video and replicated the movements. The dance videos for the intervention group were created using the following materials and procedures. One of the authors (MSakairi), with 29 years of extensive experience in dance, developed a dance program based on street dance, with reference to instructional videos for school classes [32]. The music used for the dance was selected from DOVA-SYNDROME [33]. The staff used exhaled-breath analysis to measure the dance activity level and create five videos ranging from 4.5 to 7 metabolic equivalent of task (METs), measuring the intensity of physical activity that represents the metabolic rate relative to the resting metabolic rate (Figure 1). The formula used to calculate METs is expressed as follows:



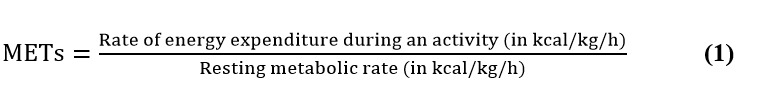



During the dance activity, METs were measured using a respiratory gas analyzer (pulmonary exercise load monitoring system: AE-310S, Minato Medical Science Co, Ltd, Osaka city, Osaka, Japan). The average METs for each dance video were as follows: (1) 4.57, (2) 4.86, (3) 4.84, (4) 6.95, and (5) 7.11 METs. Measurements were conducted using the breath-by-breath method to calculate VO_2_ and VCO_2_ based on signals from high-precision flow sensors [34]. We uploaded the created dance videos to YouTube with restricted access.

#### Intervention Group Procedures

On the day of recruitment, we provided the intervention group with a URL to access the five YouTube videos. Participants were instructed to freely select a dance from the 5 videos and perform it daily while watching the video. We did not provide any guidance on dance instruction or supervision during the dance sessions. However, we instructed the control group to freely select any exercise other than dance and perform it for 10 minutes daily. Additionally, on the day of recruitment, BP and body composition were measured, and web-based surveys were administered using Google Forms to all participants. BP was measured using an automatic medical electronic BP monitor (HBP-9035 Kentaro, OMRON Health Care Co, Ltd, Kyoto City, Kyoto Prefecture, Japan).

Participants from both groups were instructed not to change their lifestyle 2 weeks from the day of recruitment and to wear an ActiGraph continuously during this period, except during sleep and bathing. ActiGraph is a 3-axis accelerometer (wGT3X-BT ActiGraph, ActiGraph, LLC). Actigraph triaxial accelerometers are the most extensively used devices in numerous studies focused on monitoring human physical activity energy expenditure; they are capable of detecting changes in motion and converting them into digital signals, which can then be analyzed to estimate energy expenditure [35].

Two weeks after recruitment, both the intervention and control groups were instructed to begin their designated exercises and continue until the end of the study period.

**Figure 2:**
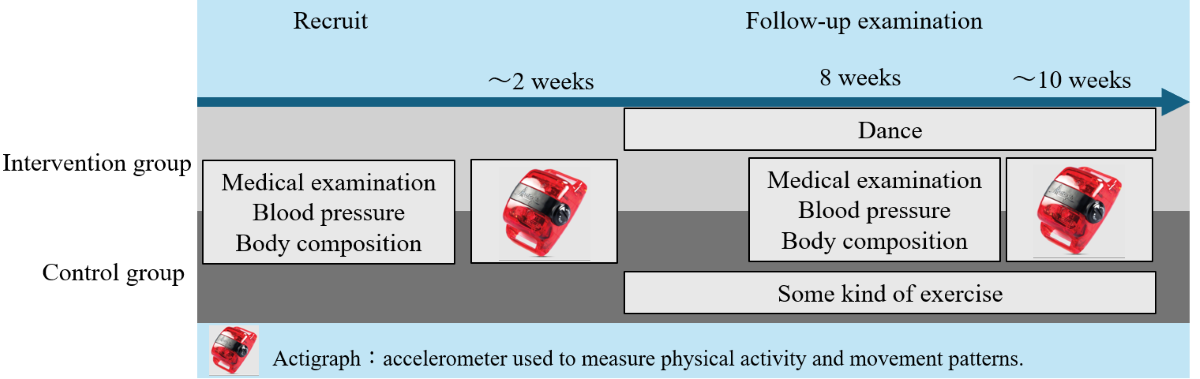
Research schedule. We instructed both the intervention group and the control group to exercise and measured their physical activity levels using an actigraph.

Approximately 2 months after recruitment, during a regular outpatient visit, BP and body composition were measured again, and another web-based survey was completed. Subsequently, the participants were instructed to wear the ActiGraph continuously, except during sleep and bathing, for another 2 weeks (Figure 2). During the intervention period, participants in both the intervention and control groups did not receive exercise guidance, nor were the frequency or manner of their exercise monitored. We did not compensate the participants of this study. The research data of patients in this study were anonymized using identification numbers; however, researchers could still identify individual patients with these numbers.

### Outcome Measures

#### Variables

The variables used in this study were gender, age, number of antihypertensive drugs, number of lifestyle-related diseases (diabetes, dyslipidemia, and hyperuricemia), medical history (cerebral infarction and ischemic heart disease), height, body weight, body muscle mass, body fat mass, family in need of care (children and adults), the presence of cohabitants, exercise habits, systolic blood pressure (SBP), diastolic blood pressure (DBP), and MVPA per day (corresponding to activity levels that are moderate or higher in intensity, namely, a level of 3 METs or higher).

#### Primary Outcome

The main outcome of this study was BP. During the study period, we measured the BP and body composition of the patients twice for comparison. This was performed on the day of participation and 2 months after participation during outpatient visits.

### Data Collection

We obtained the participants’ gender, age, frequency of antihypertensive medication use, lifestyle-related diseases (diabetes, dyslipidemia, and hyperuricemia), and medical history (cerebral infarction, and ischemic heart disease) from medical records for both groups. The body composition measured on the day of recruitment and 2 months later included height, weight, muscle mass, and body fat mass. In addition, a web-based survey using Google Forms was conducted to inquire about the presence of cohabitants, caregivers (both children and adults), and exercise habits. The criteria of the ActiGraph for adopting the data involved confirming valid days with worn durations of 10 hours or more per day, with at least 7 such days within 2 weeks. The average value for the adopted days was calculated for each individual [36-38]. In this study, as it is exploratory research rather than a confirmatory study, we did not perform multiplicity adjustments.

### Statistical Analyses

All statistical analyses were performed using JMP Pro (version 16.0; SAS Institute). All reported *P* values were 2-tailed, and *P* values <.05 were considered statistically significant. The results are presented as mean (SD) for continuous variables or as prevalence (%) for categorical variables. Comparisons between two groups were performed using the chi-square test. Multiple regression analysis was performed on both groups, with BP as the dependent variable. The other covariates were gender, age, and daily MVPA before starting exercise.

## Results

**Figure 3:**
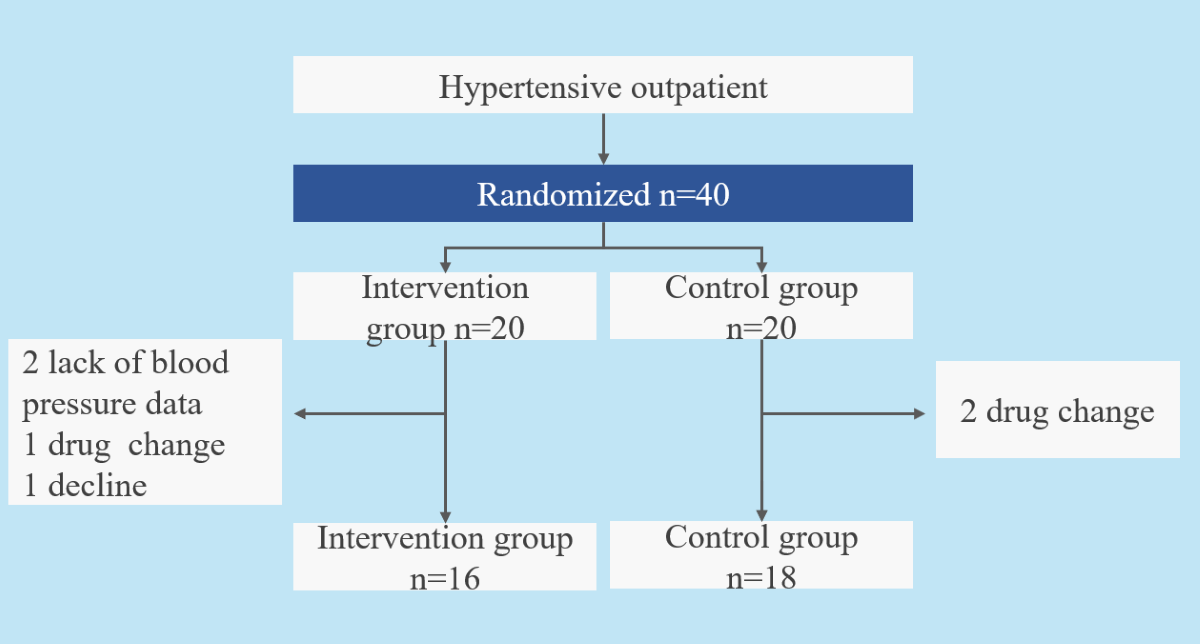
Number of participants and exclusions from the study. Four participants were excluded from the intervention group and two from the control group.

A total of 40 patients participated in the study (see Multimedia Appendix 1 for CONSORT [Consolidated Standards of Reporting Trials] checklist), and 20 outpatients were evaluated in each intervention and control group. We excluded 2 patients who lacked BP data, one patient who changed medications, and 1 patient who withdrew to care for a parent from the dance group. We also excluded one patient who changed medications and one patient who took a double dose from the control group. These participants could have experienced BP changes due to antihypertensive medications, and the lack of BP data makes evaluation difficult. Including these participants may reduce validity, so it is reasonable to exclude them. Therefore, 16 patients in the intervention group and 18 patients in the control group were analyzed (Figure 3). Among the participants, 18 (53%) participants were female, 4 (12%) participants were family caregivers, and 19 (56%) participants had lifestyle diseases (diabetes, dyslipidemia, and hyperuricemia). The mean age was 56 (SD 9.8) years, the mean number of patients who took treatment with an antihypertensive drug was 1.5 (SD 0.5), the mean BMI was 28.0 (6.3) m/kg^2^, the mean body muscle mass was 46.5 (SD 9.6) kg, the mean body fat mass was 25.3 (SD 13.8) kg, the mean MVPA time of per day was 20.8 (SD 14.3) minutes, and the mean SBP and DBP were 139.5 (SD 17.1) and 85.8 (SD 9.1) mm Hg (Table 1).

**Table 1:** Characteristics comparing intervention and control groups^a^.

Variable	Total (n=34)	Intervention group (n=16)	Control group (n=18)	*P* value
Sex (female), n (%)	18 (53)	9 (56)	9 (50)	.70
Age (years), mean (SD)	56 (9.8)	54 (11)	59 (8)	.20
Antihypertensive drug, mean (SD)	1.5 (0.5)	1.5 (0.5)	1.5 (0.5)	.10
Lifestyle disease, n (%)	19 (56)	8 (50)	11 (61)	.50
BMI (m/kg^2^), mean (SD)	28.0 (6.3)	27.2 (1.5)	29.1 (1.5)	.80
Body muscle mass (kg), mean (SD)	46.5 (9.6)	45.4 (9.7)	47.4 (9.7)	.60
Body fat mass (kg), mean (SD)	25.3 (13.8)	23.0 (14.0)	27.1 (13.9)	.40
Family caregiver, n (%)	4 (12)	2 (13)	2 (11)	.90
SBP^b^ (mm Hg), mean (SD)	139.5 (17.1)	141 (4.6)	138.2 (3.8)	.60
^c^DBP (mm Hg), mean (SD)	85.8 (9.1)	86.3 (11.4)	85.4 (6.8)	.80
MVPA^d^ per day (minutes), mean (SD)	20.8 (14.3)	24.7 (4.6)	17.3 (9.7)	.20

^a^This is the blood pressure measured on the first day of recruitment.

^b^SBP: systolic blood pressure.

^c^DBP: diastolic blood pressure.

^d^MVPA: moderate to vigorous physical activity (moderate intensity activities range from 3.0 to 5.9 METs, while high-intensity activities are 6.0 METs or above).

As a result, there was a difference in SBP between the 2 groups. The mean for the intervention group was –7.9 (SD 18.1) mm Hg and the mean for the control group was 3.9 (SD 14.5) mm Hg (*P*=.04). No difference was observed in DBP (mean –6.6, SD 11.1 mm Hg; mean –0.94, SD 10.6 mm Hg; *P*=.14), body weight (mean –3.5, SD 13.3 kg; mean –5.4, SD 18.7 kg; *P*=.74), body muscle mass (mean –7.9, SD 16.6 kg; mean –5.1, SD 15.6 kg; *P*=.61), body fat mass (mean –0.075, SD 1.1 kg; mean –1.0, SD 0.46 kg; *P*=.06), time of MVPA (mean 1.4, SD 7.5 min; mean –1.1, SD 6.9 min; *P*=.32) between the group and control group (Table 2).

**Table 2:** Amount of change before and after intervention between groups^a^.

	Systolic blood pressure				Diastolic blood pressure		
	Estimate	SD	*P* value	Estimate		SD	*P* value
Dance	–12.8	6.1	.047	–9.7		3.3	.006
Sex	–2.8	5.9	.60	–1.1		3.1	.70
Age	–0.5	0.3	.10	–0.6		0.2	.001
Pre-MVPA^b^ (minutes)	–0.2	0.2	.30	–0.006		0.1	.09

^a^Missing values were excluded from the analysis.

^b^SBP: systolic blood pressure.

^c^DBP: diastolic blood pressure.

^d^MVPA: moderate to vigorous physical activity.

In the multivariate analysis, SBP and DBP improved significantly in the intervention group compared with the control group (mean SBP –12.8, SD 6.1 mm Hg; *P*=.05; mean DBP 9.7, SD 3.3 mm Hg; *P*=.006). For the other covariates, only age showed a significant difference in DBP (*P*=.001; Table 3). No significant harm or unexpected effects were reported during this study.

**Table 3:** Multivariable analysis of systolic/diastolic blood pressure and each response variable^a^.

	Systolic blood pressure			Diastolic blood pressure		
	Estimate	SD	*P* value	Estimate	SD	*P* value
Dance	–12.8	6.1	.047	–9.7	3.3	.006
Sex	–2.8	5.9	.60	–1.1	3.1	.70
Age	–0.5	0.3	.10	–0.6	0.2	.001
Pre-MVPA^b^ (minutes)	–0.2	0.2	.30	–0.006	0.1	.09

^a^Missing values were excluded from the analysis.

^b^MVPA: moderate to vigorous physical activity.

## Discussion

### Principal Findings

Our results confirmed that regular exercise therapy using dance videos can lower the BP of patients with hypertension, even without monitoring. To the best of our knowledge, this is the first report of this finding.

BP control is crucial to maintaining health. However, various barriers, such as environmental and time constraints, prevent patients from engaging in exercise, which is a useful nonpharmacological therapy for BP control.

### The Relationship Between Exercise and BP

Regarding the relationship between exercise and BP, the antihypertensive effects of aerobic exercise have been well documented in numerous meta-analyses [8,39,40]. Aerobic exercise can significantly decrease SBP and DBP, with specific reductions observed in postmenopausal women and those who participate in combined aerobic and resistance exercises [41]. The American College of Cardiology/American Heart Association guidelines report that exercise therapy can reduce SBP by 2-5 mm Hg and DBP by 1-4 mm Hg [42]. An 8-week stepping exercise program lowered SBP/DBP by 13.1/14.8 mm Hg in older women with stage 1 hypertension [43]. In another study, swimming reduced SBP and DBP by 9 mm Hg over 20 weeks [44]. A meta-analysis of 22 trials (736 participants) examining the effects of regular running on resting BP showed a significant reduction in hypertensive patients’ resting BP, with a weighted MD of SBP –5.6 mm Hg (95% CI –9.1 to –2.1; *P*=.01) and DBP –5.2 mm Hg (95% CI –9.0 to –1.4; *P*<.01) [11]. A meta-analysis of 32 studies examining the effects of walking interventions on cardiovascular disease risk factors found a significant improvement in BP among patients with hypertension, with SBP –3.58 mm Hg (95% CI –5.19 to –1.97) and DBP –1.54 mm Hg (95% CI –2.83 to –0.26) [45]. Although the mechanisms underlying these effects are not fully understood, several other factors have been considered. Exercise likely reduces arterial pressure by decreasing cardiac output and total peripheral resistance [46]. Exercise reduces vascular responsiveness to norepinephrine, which increases vascular resistance, and reduces plasma endothelin-1 concentration. Furthermore, endothelium-dependent vasodilation is critically dependent on the production of nitric oxide. Exercise training has been shown to increase nitric oxide production and improve vasodilatory function in healthy participants [47-58]. Vertical head movements during moderate exercise may reduce angiotensin II type 1 receptor expression and BP [59]. Other mechanisms include structural changes in the blood vessels and genetic factors; however, more data are needed [60-62]. In this study, the dance group showed significant improvement in SBP and DBP compared to the control group (mean SBP –12.8, SD 6.1 mm Hg and mean DBP –9.7, SD 3.3 mm Hg). This improvement is comparable to that observed with other aerobic exercises.

### The Relationship Between Dance and BP and Monitoring Methods in Previous Studies

Dance is a dynamic aerobic endurance exercise that is broadly defined as moving one’s body rhythmically to music, usually as a form of artistic or emotional expression. Many health benefits of dance have been realized in recent years. In a previous meta-analysis, the effects of dancing on a large variety of physical health measures were assessed in healthy adults. Studies on healthy adults have found that dance is equal to or greater than exercise in terms of its effectiveness in improving physical health [63-68]. Additionally, a meta-analysis comparing dance with other exercises showed that attrition rates from dance interventions were reported to be lower or equal to exercise, and adherence rates from dance interventions were higher or similar to exercise [21]. In a meta-analysis, dance therapy significantly reduced BP in patients with hypertension, with reductions of approximately 12 mm Hg in SBP and 3.4 mm Hg in DBP [69]. Patients with hypertension undergoing dance movement therapy experience reductions in SBP by 19.2 mm Hg and DBP by 9.5 mm Hg after 4 weeks of twice-weekly sessions [25]. Dances performed in dance movement therapy are often rooted in modern dance [26], but other dance genres also have a positive impact on BP control in patients with hypertension. In aerobic dance, participants saw a decrease in SBP by 18.8 mm Hg and DBP by 8.9 mm Hg over 12 weeks of 45-minute sessions three times a week [27]. Hula dance participants experienced a reduction in SBP by 18.3 mm Hg compared to 7.6 mm Hg in the control group after 12 weeks of 60-minute sessions twice a week [28]. In a study of older adults performing folk dance, SBP decreased from 146.8 mm Hg to 133.8 mm Hg and DBP from 78 mm Hg to 72 mm Hg over 12 weeks of 50-minute sessions three times a week [29]. Additionally, chain dance led to a decrease in SBP by 9 mm Hg and DBP by 6 mm Hg after 6 weeks of 30 to 45-minute sessions twice a week [30]. Overall, dance has been suggested to be highly effective in improving BP, and the results of this study support this.

### Differences Between Previous Dance Studies and Ours

Naturally, exercise prescriptions are meaningless unless implemented by patients. The method of monitoring exercise implementation is likely an important factor in evaluating the effectiveness of exercise therapy in patients with hypertension. In previous studies investigating the relationship between dance exercise prescriptions and BP control, improvements in BP control were observed in all cases. However, as mentioned, in all these studies, the execution of dance exercises was monitored face-to-face or through other means. The most significant difference between this study and the previous research is that we tested the effectiveness of dance-based exercise prescriptions on BP without monitoring. To our knowledge, no previous study has examined the antihypertensive effects of dancing without monitoring. This study is the first to entrust everything to the patients themselves, without monitoring whether the exercise prescriptions were carried out or how accurately the participants performed the dance. In this study, we did not conduct monitoring during the dance sessions; the SBP and DBP in the dance group showed a significant improvement compared with those in the control group. General outpatient care must be carried out in a very short time, lasting only 5-10 minutes, and the existence of a fixed tool that can be used without supervision is thought to be highly effective in the management of lifestyle-related diseases.

Therefore, dance exercises using dance videos may be superior to other forms of exercise in terms of sustainability. Previous noninterventional studies have found that the primary intrinsic motivator for participation in dance was having fun [70] or improving mood [71], whereas participants also experienced significant physical benefits. This was a secondary motivator for initial and maintained participation, thereby likely demonstrating the enjoyment and adherence link that exists in dance. It is presumed that the pleasure and enjoyment experienced by many through dance offers the additional advantage of an increased likelihood of regular participation and adherence, which are essential features for achieving long-term health benefits and could explain the results seen in the included studies. This result is consistent with previous findings. Additionally, in this study, a dance exercise video posted on YouTube was provided as reference material for physical activity. This approach may have facilitated patients’ access to an exercise “model,” potentially leading to improved adherence to the prescribed physical activity.

### The Significance of Applying This Study to Clinical Medicine

Incorporating exercise prescriptions using YouTube dance exercise videos into outpatient treatment may improve BP control in patients with hypertension, similar to other exercise prescriptions, even in busy and understaffed outpatient settings without monitoring. If video-based dance prescriptions, such as those used in this study, were put into practice, doctors would only need to provide patients with dance prescription videos. This could eliminate the need to spend valuable time during outpatient visits explaining exercises or monitoring exercise routines.

### Limitations

This study had a few limitations.

First, because the patients were recruited from a single university hospital, there may be a risk of selection bias. In the future, this can be improved by recruiting more participants from additional outpatient clinics.

Second, the frequency of dance sessions and the accuracy of movements in the intervention group were unknown. Exercise therapy, intensity, and duration in the control group were also unknown because they were not measured.

Third, the timing of the outpatient visit was generally set at 8 weeks after registration for both BP and body composition measurements; however, there was some variation due to the timing of the outpatient visit.

Fourth, factors such as exercise, diet, and sleep immediately before BP measurement were not standardized because the schedule was adjusted to suit the participants’ convenience.

Fifth, since three participants from each group dropped out during the observation period, BP changes in these individuals may have occurred due to antihypertensive medications, making evaluation difficult due to the absence of BP data. Including these participants could reduce the validity of the study; therefore, their exclusion is appropriate.

Despite these limitations, this study remains useful, though it faces constraints due to its focus on verifying the effectiveness of exercise prescriptions through dance videos in outpatient settings.

### Conclusions

This study examined the effects of videos of unsupervised dance exercises on patients with hypertension. The results showed that dance videos were more effective in lowering BP than conventional exercise prescriptions. These results will contribute to exercise therapy for patients with lifestyle-related diseases.

## Appendix

Multimedia Appendix 1 CONSORT-eHEALTH checklist (V 1.6.1).

## Abbreviations

BP: blood pressure
CONSORT: Consolidated Standards of Reporting Trials
DBP: diastolic blood pressure
MD: mean difference
MET: metabolic equivalent of task
MVPA: moderate to vigorous physical activity
SBP: systolic blood pressure
UMIN: University Hospital Medical Information Network

## Data Availability

The datasets generated or analyzed during this study are available from the corresponding author upon reasonable request.
